# Climate change, health, and conflict in Africa’s arc of instability

**DOI:** 10.1177/17579139211058299

**Published:** 2021-11-17

**Authors:** MS Evans, B Munslow

**Affiliations:** Centre for Environmental Health and Sustainability, University of Leicester, Leicester LE1 7RH, UK; Liverpool School of Tropical Medicine, Liverpool, UK

**Keywords:** environment, climate change, global health

## Introduction

**Figure 1:**
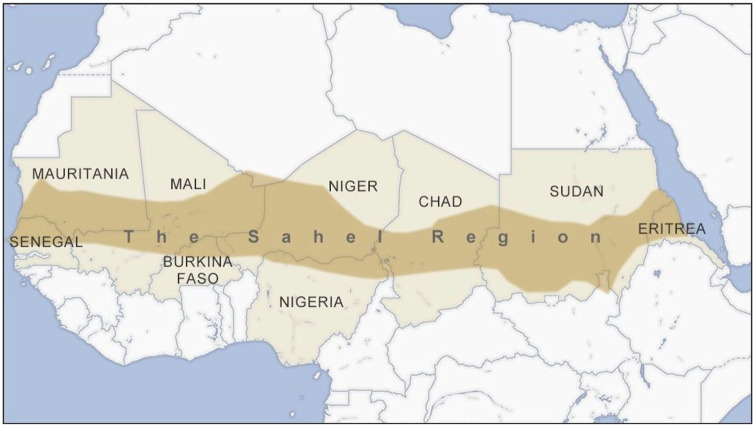
Africa’s arc of instability follows the Sahel region^4^

Climate change lies at the heart of many complex humanitarian emergencies and emerging global health challenges, with Sub-Saharan Africa (SSA) identified as one of the most vulnerable regions to the impacts of climate change.^1^ An arc stretching from Somalia and Eritrea in the east to Mauritania in the west forms a band of countries that are particularly vulnerable to the consequences of climate change (Figure 1).^2^ This arc experiences a devastating combination of state fragmentation, Islamist insurgency, and climate change, undermining livelihood strategies across the region. A climate injustice exists; despite contributing relatively little to the anthropogenic causes of climate change, individuals living in these countries face the most severe impacts.^3^

## Context: Climate Change and Health

**Figure 2:**
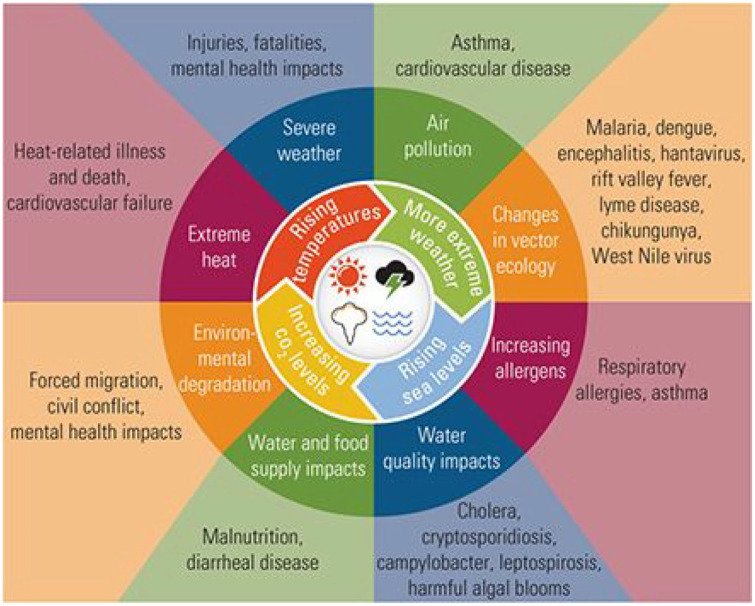
Potential consequences of climate change^15^

The consequences of climate change are diverse, severe, and predicted to worsen over the coming years (Figure 2). Even if temperature changes are maintained in line with the Paris Agreement (that is to limit temperature increases to below 2°C, and preferably below 1.5°C, compared to pre-industrial levels), there will be significant impacts on biodiversity, water availability, food security, and health.^5^

In SSA, extreme weather events including storms, floods, droughts, and heatwaves are increasing in frequency, influencing health in a number of ways.^5^ Although the epidemiological distribution of specific infectious diseases varies dependant on organism and context, in general, increased temperature and extremes of precipitation have been associated with increased risk of diarrhoeal illness,^6^ which is a leading cause of mortality in Africa.^7^ Vector-borne diseases, which are especially sensitive to changes in climatic and weather conditions, represent a particular cause for concern. Dengue, transmitted by *Aedes* mosquitoes, is one of the fastest spreading infectious diseases and has been identified by the World Health Organization (WHO) as one of the top 10 threats to global health.^8^ Temperature changes mean dengue incidence is predicted to increase across large parts of SSA and may eventually contribute a greater burden of disease than malaria.^9^

Changing climate and weather events affect regional food security. Yields of crops such as wheat and maize have been adversely affected by climate change in many lower-latitude areas.^10^ This is predicted to worsen over the next 50 years, yet the European Commission report that 37 million people in the Sahel region are already severely or moderately food insecure.^11^ Pastoralism represents a common livelihood strategy in countries along the arc; these systems are extremely vulnerable to the effects of climate change. Droughts and floods have disrupted livelihoods and decimated crops and livestock.^12^ Dwindling natural resources, decreased animal and pasture productivity, and loss of biodiversity have contributed to rising pressure in the region, exacerbating underlying social and political tensions and at times erupting into violent conflict.

As a consequence of these factors, many millions of people living along the arc have been forced to migrate. Earlier this year, the United Nations High Commissioner for Refugees (UNHCR) reported that some 2 million people have been displaced within the Sahel region.^13^ Many seek shelter within neighbouring countries, while others attempt dangerous sea journeys to Europe. The displacement of populations has the potential to spread infectious diseases into previously immuno-naïve areas and expanding refugee camps can also lead to outbreaks of diseases. There is increasing recognition of the impact of forced migration and the use of refugee camps on mental health and of the possibility of sexual assaults.^14^ However, these issues are not frequently acknowledged as potential consequences of climate change.

We can best illustrate the issues described above by first focusing narrowly on one region of a country affected, Darfur in Sudan. Following this, we consider the broader view by examining the similarities of three Sahelian countries in the arc of instability: Mali, Niger, and Burkina Faso.

## Darfur

‘*Darfur’s landscapes have a cruel beauty’*.^16^ Darfur, in western Sudan, has been heavily afflicted by both climate change and conflict. Some consider the violence in the region to represent the first genocide of the 21^st^ century, others suggest it embodies the first climate change war.^17^ Over the last 40 years, conflict in the region has claimed millions of civilian lives and displaced many more.^17^ Reasons for the violence are complex, involving an explosive combination of political, social, and environmental factors.

Darfur contains several distinct geographical and climatic zones within which different ethnic groups and livelihood strategies predominate. The edge of the Sahara and the Sahel deserts lie in the northern region of Darfur, where nomadic pastoralism dominates. In the south, crop farming constitutes the main livelihood strategy as the rich, alluvial soil provides ideal conditions for farming. Between the two zones lies a semi-arid region dominated by the Jebel Marra mountains. Migration, intermarriage, and nomadic lifestyles have led to a plurality of cultures and a blurring of ethnic groups between these areas.^18^

Nomadic pastoralists from the north have long exerted traditional rights to migrate south for water and grazing land for their cattle, extending into farming areas. However, climate change has led to rising temperatures, desertification, and unpredictable weather patterns affecting crop yields. ‘*In recent years drought, desertification and soil loss have seen the Sahara creep south into the Sahel, and the Sahel in turn creep south into the Sudanian Savanna*’.^19^ Nomadic pastoralists continue to exert their traditional rights, but now there is a scarcity of water and pasture leading to competition over reduced resources and rising tensions between communities.^18^

There is debate over whether climate change has precipitated the conflict in Darfur or whether it has exacerbated pre-existing tensions in the region, acting as a ‘force-multiplier’.^20^ In Darfur, the role of ethnicity is closely woven with that of livelihoods. Predominantly, pastoralists are of Arabic ethnicity, while agriculturalists are mostly ethno-African. The precarious relationship between pastoralist and farmer is gradually eroded, creating tension and violence between groups.^16^

## Mali, Niger, and Burkina Faso

These three Sahelian countries have become the epicentre of a Jihadi upsurge since 2012 with climate change acting as a powerful compounding factor: weakening livelihoods place pressure on economic and social systems with radicalising political ramifications. The volume of the Niger River has shrunk by a third over the past three decades.^21^ Usable arable land is declining as the population increases.^22^ This obliges many farmers to no longer use fallow, interrupting the historical passage across their land by herders of cattle after the harvest; now the herders destroy crops on the old routes and also change migration patterns impacting other farmers.^23^ Governments favour farmers over herders, creating a pastoralist recruiting ground for Jihadis, such as among the Tuareg and Fulani populations.^24^ In essence, climate change is facilitating the fusing of pastoralist grievances with Jihadi politics.^25^

Weak states, artificial frontiers, and pastoralist livelihood systems facilitate militia groups spreading their influence across the borders of the three states.^26^ In the Western Sahel in 2019, attacks increased 86% over the previous year causing 5000 deaths with 5.1 million needing humanitarian assistance in the form of nutrition, health care, and shelter.^27^ Weak states leave a vacuum in the periphery, worsened by a failed decentralisation strategy which decimated services and security.^28^ As rebel forces gained recruits, military retaliation on local communities created further disaffection,^29^ aiding Jihadi recruitment of the Fulani. Jihadists offer a rule of law where none exists, and an alternative livelihood strategy where climate change and misgovernment have taken away other options.

## Conclusion

Climate change is exerting a multiplier impact on health challenges and conflict in the Sahel region. An arc of instability has been created stretching from the Atlantic to the Red Sea and Indian Ocean. Many health issues can only be tackled in the long term if climate change adaptation and mitigation globally become a priority. Humanitarian healthcare delivery, currently under attack,^30^ will need to be strengthened and supported in the Sahel region. As livelihood strategies are obliged to change, poverty alleviation efforts are also needed to reduce the human costs of Jihadist violence and government repression. Forced migration currently from the Sahel region has widespread global ramifications.
